# NEW ACCELEROMETRY PATTERNS IN FRAILTY: HOURLY ACTIVITY AND VARIANCE.

**DOI:** 10.1093/geroni/igz038.3063

**Published:** 2019-11-08

**Authors:** Megan Huisingh-Scheetz, Kristen Wroblewski, Linda Waite, Elbert Huang, Donald Hedeker, L P Schumm

**Affiliations:** University of Chicago, Chicago, Illinois, United States

## Abstract

Wearable sensors may improve our ability to identify frailty in the community. Frailty has been historically defined, in part, by reduced average activity; however, new analytic methods of aggregate, free-living accelerometry data suggest that frailty may be more fully characterized above and beyond reduced average activity. Using mixed-effect regression models of awake hourly activity from the National Social Life, Health and Aging Project dataset, we have shown that frail adult activity is most reduced in the morning relative to pre- and non-frail adults rather than the afternoon or evening. High residual between- and within-subject activity variance in this model prompted further study of activity variance. A follow-up analysis using a mixed-effect location-scale model of hourly activity data revealed that increasing frailty in older adults is associated with greater between-subject as well as within-subject hourly activity variability, particularly in the morning and afternoon. Study implications and future directions will be discussed.

